# Everybody's Page

**Published:** 1892-12-31

**Authors:** 


### EVERYBODY'S PAGE.

According to a writer in the *Annales d'Hygiène* material prosperity and height of stature are closely associated. In the poorer provinces of France men are found to be shorter than in the more prosperous regions. This fact will no doubt be accounted for in some measure by the better nourishment received in the latter.

There seems to be a craze with some people for making experiments on animals, which common sense should tell them can have no useful result. A very painful instance of this was shown recently abroad. A less harmful but not less useless experiment is alleged to have been made on a dog in order to ascertain whether exercise taken immediately after food is prejudicial. The rudiments of logic might have saved him the trouble of his experiments. Though men and dogs are animals, their respective conditions of life are widely different. Most of us are taught in a manner as practical as unpleasant that exercise taken immediately after food is injurious to health. We congratulate the experimentalizer on not having made the discovery for himself.

The dangers surrounding modern life are rapidly becoming almost insupportable, and it is really unkind to disclose new ones. The milk-can, so familiar to view at our railway stations and in our milk carts, is the latest addition. We are now told that milk cans are dangerous to health, owing to their frequently being uncleanly and open to the admission of impure air. But the uncleanliness is not a necessary adjunct to the milk-can as it is; it might be the unwelcome inmate of the most scientifically as well as of the most unscientifically constructed can. Our forefathers, if they could visit us and be told of the dangers they passed through in their pre-sanitary days, would wonder how they ever reached manhood, and it is just possible they might think some of our supposed dangers were mares' nests.

Medicine as a profession is not an avenue in Berlin, any more than it is elswhere, to immediate affluence or even prospective wealth in the most remote future, except to a favoured few. According to recent Income Tax returns out of nearly eighteen hundred doctors, half make incomes of less than £150 a-year, two hundred and fifty only make £400 a-year and not more than one hundred and seventy make over £500 a-year. This is a poor reward for a laborious life.

So many injuries to health have happened and are constantly happening from the eating of tinned meats and fish, that some of the great Canadian and American companies have decided to substitute glass jars for tins hitherto used. The wisdom of this decision is questionable, seeing that a new danger is substituted for an old one in the form of liability to injury from chipped pieces of glass, as proved by the use of glass jars for jams. Would not stoneware prove more suitable for all such purposes?

In his book on the "Alpine Winter in its Medical Aspects," Dr. Tucker Wise gives some reasons for the superiority of Continental health resorts over English ones which are open to controversy, however valid they may have been a few years ago. He maintains that the treatment of icipient consumption in resorts of the South of England is so unsatisfactory owing to the fact that little or no attention is paid to the requirements for comfort and health of invalids. There are scarcely any shelters provided —no glass-covered galleries, verandahs, nor convenience for taking the air in wet weather. Indoors the temperature maintained by stoves and open fires is most uncertain and partial. This is rather hard upon some of our health resorts, where a good deal has been done of late years to temper the wind to the shorn lamb.

